# *In vitro* biological control of *Pyrrhoderma noxium* using volatile compounds produced by termite gut-associated streptomycetes

**DOI:** 10.3389/fpls.2024.1371285

**Published:** 2024-03-04

**Authors:** Cherrihan Adra, Harrchun Panchalingam, Keith Foster, Russell Tomlin, R. Andrew Hayes, D. İpek Kurtböke

**Affiliations:** ^1^ School of Science, Technology and Engineering, University of the Sunshine Coast, Sippy Downs, QLD, Australia; ^2^ Brisbane City Council, Program, Planning and Integration, Brisbane Square, Brisbane, QLD, Australia; ^3^ Forest Industries Research Centre, University of the Sunshine Coast, Sippy Downs, QLD, Australia

**Keywords:** *Streptomyces*, streptomycetes, termites, termite gut symbiosis, antifungal compounds, volatile organic compounds, biological control, bio-fumigation

## Abstract

**Introduction:**

*Pyrrhoderma noxium* is a plant pathogen that causes economic losses in agricultural and forestry industries, including significant destruction to amenity trees within the city of Brisbane in Australia. Use of chemical control agents are restricted in public areas, there is therefore an urgent need to investigate biological control approaches. Members of the phylum Actinomycetota, commonly known as actinomycetes, are known for their industrially important secondary metabolites including antifungal agents. They have proven to be ideal candidates to produce environmentally friendly compounds including the volatile organic compounds (VOCs) which can be used as biofumigants.

**Methods:**

Different *Streptomyces* species (n=15) previously isolated from the guts of termites and stored in the University of the Sunshine Coast’sMicrobial Library were tested for their antifungal VOCs against *Pyrrhoderma noxium*.

**Results:**

Fourteen of them were found to display inhibition (39.39-100%) to the mycelial development of the pathogen. Strongest antifungal activity displaying isolates USC-592, USC-595, USC-6910 and USC-6928 against the pathogen were selected for further investigations. Their VOCs were also found to have plant growth promotional activity observed for *Arabidopsis thaliana* with an increase of root length (22-36%) and shoot length (26-57%). The chlorophyll content of the test plant had a slight increase of 11.8% as well. Identified VOCs included geosmin, 2-methylisoborneol, 2-methylbutyrate, methylene cyclopentane, β-pinene, dimethyl disulfide, ethyl isovalerate, methoxyphenyl-oxime and α-pinene. Additionally, all 15 Streptomyces isolates were found to produce siderophores and indole acetic acid as well as the enzyme chitinase which is known to break down the fungal cell wall.

**Discussion:**

Findings indicate that termite gut-associated streptomycetes might be used to control *Pyrrhoderma noxium* by utilizing their wide range of inhibitory mechanisms.

## 1 Introduction

Phytopathogens pose a significant threat to global cultivation of economically and culturally important plant species (Boukaew et al., 2021), causing losses estimated at around 30-40% and costing $200 billion (US) annually (Pacios-Michelena et al., 2021). One such pathogen is *Pyrrhoderma noxium*, formerly known as *Phellinus noxius*, which causes extensive damage to many fruit, ornamental, herbaceous and coniferous trees in Taiwan (Ann et al., 2002), Mariana Islands (Hodges and Tenorio, 1984), Ishigaki Island (Hattori et al., 1996), Malaysia (Mohd Farid et al., 2005) and the Ryukyu Islands of Japan (Sahashi et al., 2012). The pathogen has also now been found to cause significant damage across the city of Brisbane in the state of Queensland, Australia. The culturally important heritage fig trees (*Ficus macrophylla*) found in many of the city’s parks and roadsides have been infected with *P. noxium* resulting in brown root-rot the pathogen induces (Adra et al., 2022; Panchalingam et al., 2022). Historically, plant protection from fungal pathogens has been achieved using chemical fungicides. For instance, chemicals found most effective against *P. noxium* include propiconazole, triadimefon and prochloraz (Tsai et al., 2005). However, more environmentally friendly, and safer options are preferred for this pathogen such as implementation of biological control methods which have successfully been demonstrated for other plant pathogenic fungi (Ayed et al., 2021; Gong et al., 2022).

Biological control refers to the control, regulation or reduction of pest populations through the means of natural enemies (DeBach and Rosen, 1991; Thambugala et al., 2020). The term biological control, also abbreviated to “biocontrol”, has become popularized within the fields of plant pathology and entomology. Antagonistic bacteria were shown to provide environmentally sound alternatives to protect plant hosts against fungal diseases (Berg and Hallmann, 2006). Some known mechanisms of antagonistic bacteria towards fungal pathogens include competition, lysis through the secretion of degradative enzymes, antibiosis and through the production of volatile organic compounds (You et al., 2019).

A potential biological control solution against *P. noxium* may be found through the investigation of actinomycetes, specifically, by the use of the members of the genus *Streptomyces* as they have been the prime producers of bioactive compounds (Kurtböke, 2012), including volatile organic compounds (VOCs). VOCs are low molecular weight, lipophilic chemicals with high vapor pressure which allows for the diffusion capacity through the atmosphere and soil (Wang et al., 2013; Lyu et al., 2020; Ayed et al., 2021). They are also ideal for crop protection as they are non-toxic and easily evaporate, therefore, bio-fumigation offers an ecologically viable and environmentally safe biocontrol option (Wu et al., 2015). VOCs produced by streptomycetes include variable class structures such as alcohols, ketones, aldehydes, terpenes, and sulfur or nitrogen- containing compounds (Gong et al., 2022). These compounds from streptomycetes might have promising implications for biological control and plant protection (Gong et al., 2022) VOCs of streptomycetes were shown to effectively control fungal diseases include the ones from *S. fimicarius* against *Peronophythora litchi* (Xing et al., 2018; Li et al., 2020), *S. yanglinensis* against *Aspergillus flavus* (Lyu et al., 2020) and *S. setonii* against *Ceratocystis fimbriata* (Gong et al., 2022). Previous studies have been conducted investigating antifungal VOCs produced by various microorganisms against *P. noxium*, however, there have been no prior studies assessing VOCs from streptomycetes against *P. noxium*, although they have shown to be effective against other fungal plant pathogens.

Streptomycetes in particular from previously unexploited environments such as the termite guts might prove to be useful for detection of novel and potent new antimicrobials (Kurtböke, 2012; Ayed et al., 2021) as mutualistic associations between insects and microorganisms were shown to be important sources for bioprospecting. Streptomycetes in particular from the termite-guts were shown to produce an array of bioactive metabolites to protect termites from invading pathogens, including pathogenic fungi (Kurtböke and French, 2007; Kurtböke et al., 2014). Additionally, *Streptomyces* spp. play an important role in aiding plant growth and development as well as helping plants to resist biotic and abiotic stresses (Li et al., 2020). They are also proven to produce plant growth hormones such as indole acetic acid (IAA) as well as stimulate plant growth through mechanisms such as siderophore production (He et al., 2022). Siderophores are small molecules which bind and transport iron; an essential nutrient for plants, making it more bioavailable. *Streptomyces* spp. are also capable of inducing plant defense through the production of enzymes such as chitinases which breaks down chitin; an important constituent of the fungal cell wall (Ekundayo et al., 2022). In this study, the diversity and biocontrol potential of VOCs produced by various termite gut-associated *Streptomyces* spp. have been investigated in their ability to control *P. noxium* also as supporters of plant growth. If proved effective, the antifungal and plant growth promoting VOCs could be effective biofumigants and may be used as biological control alternatives for plant protection against crop pathogens within agricultural systems.

## 2 Materials and methods

### 2.1 Cultures and culture media

From the collection of actinomycetes that were previously isolated from the guts of termites, *Coptotermes lacteus* (Froggatt) (Kurtböke and French, 2007; 2008) and cryogenically preserved at the University of the Sunshine Coast’s Microbial Library, the 15 of them were selected to determine their antifungal VOCs. Identification and bioactive compound production by these isolates were previously examined (Adra et al 2023).

The isolates were grown on oatmeal agar (Williams and Wellington, 1983) and *Pyrrhoderma noxium* which was previously isolated from infected heritage fig trees in Brisbane by the University of Queensland researchers grown on Potato dextrose agar (PDA) (OXOID, Australia).

### 2.2 Antifungal activity of the VOCs produced by streptomycetes against *P. noxium*

**Figure 1:**
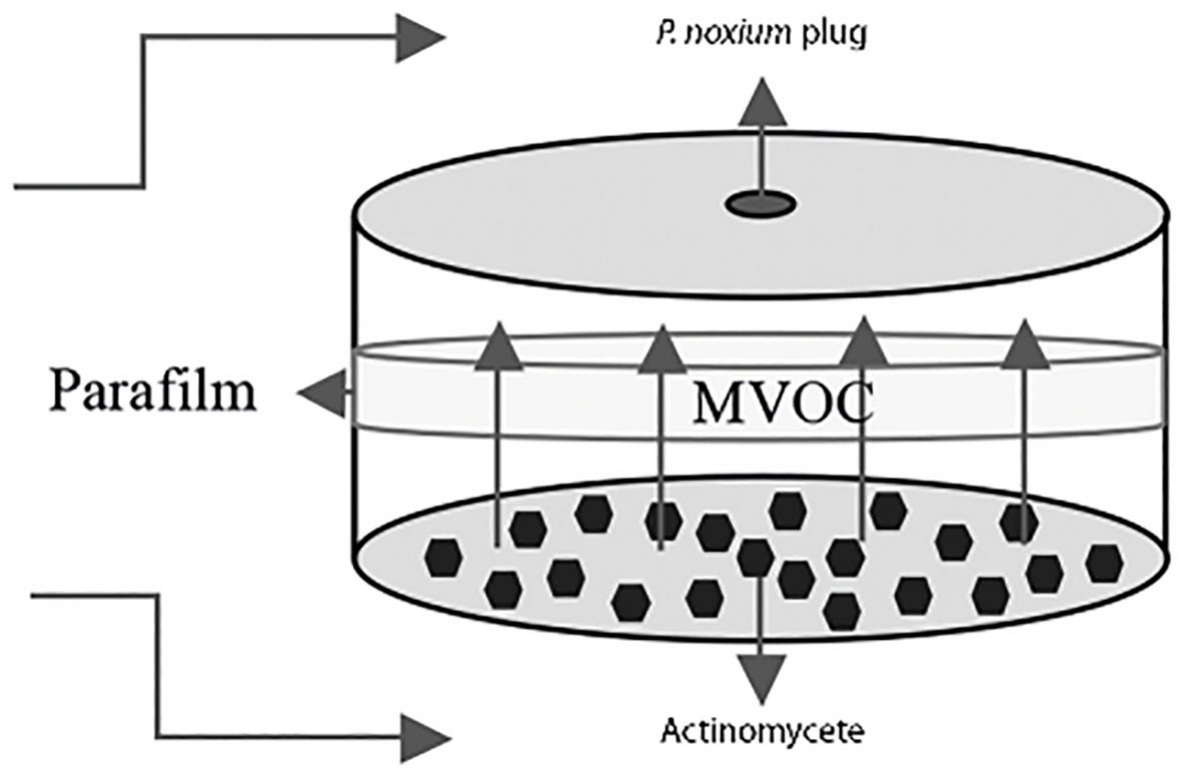
Diagram of the sandwich plate method used for the detection of inhibitory effects of VOCs produced by actinomycetes on *P. noxium.* The microbial VOCs (MVOC) interaction zone is shown in the center between the two plates.


The sandwich plate method (**Figure 1**) (Yang et al., 2018) was used to determine volatile compound(s) production capacity of the selected streptomycetes. Oatmeal agar plates were inoculated with the streptomycetes and the plates were incubated for 7 days at 28°C until they formed a confluent lawn of growth. After the 7-day incubation period, a *P. noxium* plug taken from a PDA plate was placed. These two plates without the lids were then paired, first by placing a piece of adhesive tape on each side of the plates to stabilize them, and then sealing with a Parafilm™ for three rounds to ensure full closure of the paired plates (Yang et al., 2018) (**Figure 1**). Controls were done by pairing the *P. noxium* plate with a blank oatmeal plate. Plates were prepared in triplicate and incubated at 28°C and colony diameter of *P. noxium* measured on the third day of the incubation when the fungal mycelium in the control plates reached the edge. The percentage inhibition was then calculated using the following formula:

$$\begin{array}{c} \text{Inhibition}\;\text{percentage}=\\ \sum^{}\left(\frac{\text{Colony}\;\text{diameter}\;\text{of}\;\text{pathogen}-\left(\text{colony}\;\text{diameter}\;\text{of}\;\text{pathogen}+\text{antagonist}\right)}{\text{Colony}\;\text{diameter}\;\text{of}\;\text{pathogen}}\right)\times 100 \end{array}$$

### 2.3 Plant promotional activity of the VOCs produced by actinomycetes on *Arabidopsis thaliana*

To determine whether the VOCs produced by these selected streptomycetes influenced plant growth, *Arabidopsis thaliana* seedlings were exposed to the VOCs emitted from the isolates. *Arabidopsis thaliana* seeds were surface sterilised as described by (Wu et al., 2019). Seedlings were washed in 95% ethanol for 5 minutes, then in 10% bleach for 7 minutes, they were then washed 5 times in sterile water before storing them at 4°C in the dark for 2 days allowing germination. The seedlings were then sown onto Murashige and Skoog medium (MSM) (Murashige and Skoog, 1962) supplemented with 3% sucrose and 0.7% agar and left to germinate for 5 days. The germinated seedlings were then placed onto a larger MSM plate (150 mm × 25 mm). The MSM plate contained two smaller petri dishes with oatmeal agar inoculated with streptomycetes (Panchalingam et al., 2022). Two small plates were selected as this makes up approximately half of the larger petri dishes. Control plates were blank oatmeal agar without the streptomycetes. The large MSM plates were then sealed with Parafilm™ and left to incubate at room temperature for 2 weeks (temperature: 22+1 °C, humidity: 65%, light: 382 lux). There was a total of 5 plates per streptomycete isolate treatment and 10 seedlings per plate. The root and shoot length of the test plants were subsequently measured as well as the chlorophyll content measured spectrophotometrically (Lee et al., 2016). The chlorophyll content was measured by pooling and grinding the shoots of the *A. thaliana* and submerging them overnight in 100 µL of 80% acetone in the dark at 4°C. The next day, the absorbance readings were measured at both 645 nm and 663 nm using an EnSipire ™ multimode plate reader (Lee et al., 2016; Panchalingam et al., 2022).

### 2.4 Headspace-solid-phase microextraction-gas chromatography-mass spectrometry analysis of the VOCs produced by streptomycetes

From the 15 streptomycete isolates displaying antifungal activity, four of the most active ones were selected for further analysis and their volatile profiles investigated using HS-SPME-GC-MS. The selected isolates were USC-592, USC-595, USC-6910 and USC-6928. Their 16rRNA oligonucleotide sequences were previously published by Adra et al. (2023). The isolates were inoculated onto slant oatmeal agar in GC-MS headspace vials (20 mL) from 7-day old streptomycetes plates and grown in co-culture and mono-culture conditions at 28°C for 7 days and 14 days (time difference to understand the changes in VOCs with time as well as their concentrations). For the co-culture vials, a fungal plug was placed next to the streaked streptomycetes. The volatiles were collected using SPME fibres (75 µm Carboxen/PDMS, Merk) which were inserted into the headspace vial and exposed to the volatiles for 15 min (in triplicate). These were thermally desorbed from the fibre through injection into the inlet port of the gas chromatograph (GC) (Agilent 6890 Series) coupled to a mass spectrometer (MS) (Agilent 5975) and fitted with a silica capillary column (Agilent, model HP5-MS, 30 m×250 µm ID×0.25 µm film thickness). Gas chromatograph conditions for acquiring data were – inlet temperature: 250°C, carrier gas: helium at 51 cm/s, split ratio 13:1, transfer-line temperature: 280°C, initial temperature: 40°C, initial time: 2 min, rate 10°C/min, final temperature: 260°C, final time: 6 min. The MS was held at 280°C in the ion source and the scan rate kept was 4.45 scans. Identities of the VOCs were assigned to peaks with respect to the National Institute of Standards and Technology mass spectral library (accessed 13 June 2021, version 2.0) (Panchalingam et al., 2022).

### 2.5 Scanning electron microscopy of *P. noxium* hyphae when exposed to the streptomycetes

The sandwich plate method demonstrated in the antifungal assays (above) were repeated for isolate USC-595B due to its observed strong activity, however, this time three 10 mm round coverslips were inserted into the agar plate containing the *P. noxium* at a 45° angle (Cross, 1989). These were incubated at 28°C for 3 days to allow the fungal mycelia to grow onto the coverslips. The coverslips were then gently liberated from the agar using sterile forceps and placed onto a glass slide. The slides were freeze-dried overnight (Thermo Savant, temperature -50°C, vacuum 878 mbar), placed onto SEM mounts and sputter coated with Au/Pd using the Quorum Q150T. The samples were then viewed under the Scanning Electron Microscope (JEOL JSM6010LA) and signs of inhibition for *P. noxium* mycelia were observed.

### 2.6 Indole acetic acid and siderophore production from streptomycetes

The production of IAA from the 15 streptomycetes was quantified following a colorimetric method, using Salkowski reagent (Salkowski, 1885; He et al., 2022). Streptomycete plugs taken from a 7-day old streptomycetes plate were grown in 10 mL of Glucose Yeast Malt extract (GYM) broth, containing 0.2% of l-tryptophan and incubated at 28°C at 120 rpm for 7 days in tissue culture flasks. Cultures were then centrifuged at 10,000 rpm for 15 min. The supernatant (1 mL) was then mixed with 2 mL of Salkowski reagent (a mixture of 0.5 M ferric chloride and 35% perchloric acid) (Salkowski, 1885) and the appearance of a pink colour was indicative of IAA production. The optical density was then read at 530 nm using a spectrophotometer (EnSpire™ multimode plate reader). Each isolate was examined in triplicate. The concentration of IAA in the culture was calculated using a standard curve of IAA (serial dilutions from 0-50 µg/mL) as described by Gusmiaty et al. (2019).

To determine the siderophore production by the isolates, Blue chrome azurol S (CAS) agar was utilised (Louden et al., 2011). The centre of the CAS agar was then inoculated with agar plugs cut from 7-day old streptomycetes lawn on oatmeal agar plates (in triplicate). The plates were incubated at 28°C for 10 days. Orange clearance zones were indicative of siderophore production on this specific medium (Khamna et al., 2009).

### 2.7 Quantification of chitinase production from streptomycetes

The chitinase activity for the 15 streptomycetes was ascertained using the dinitrosalicyclic acid (DNS) method by measuring the production of N-acetyl-D-glucosamine (NAGA). The procedure was modified from (Liu et al., 2015). 25 mL of 1% chitin broth was inoculated with streptomycetes from a 7-day old streptomycetes plate. These were then incubated at 28°C at 150 rpm for 14 days (measurements were taken at 7 and 14^th^ days). After incubation, 200 µL of the filtered broth (completed under vacuum) was added to 0.6 mL of DNS and 200 µL of phosphate buffer. This was heated at 37°C for 1hr using a heating block and immediately transferred to 100°C for 1 hr. The samples were rapidly cooled to room temperature and centrifuged at 13, 500 g for 5 min. The absorbance of the supernatant was measured at 540 nm with one unit of chitinase activity (U) being defined as the amount of enzyme releasing 1 µmol of NAGA per minute under standard assay conditions.

### 2.8 Statistical analysis

Statistical analysis was performed using SPSS software version 29.0. Prior to the analysis, the normality of data distribution was assessed using the Shapiro-Wilk test and the homogeneity of variances was determined using the Levene’s test. One-way ANOVA was conducted to test significant differences between the means. When homogeneity of variance was demonstrated, *post-hoc* pairwise comparisons were performed using Tukey’s test. However, where homogeneity of variance was not demonstrated, the Games-Howell test was used. All statistical analyses were performed at a significance level of α = 0.05.

## 3 Results

### 3.1 Antifungal activity of the VOCs produced by streptomycetes’ VOCs against *P. noxium*

**Figure 2:**
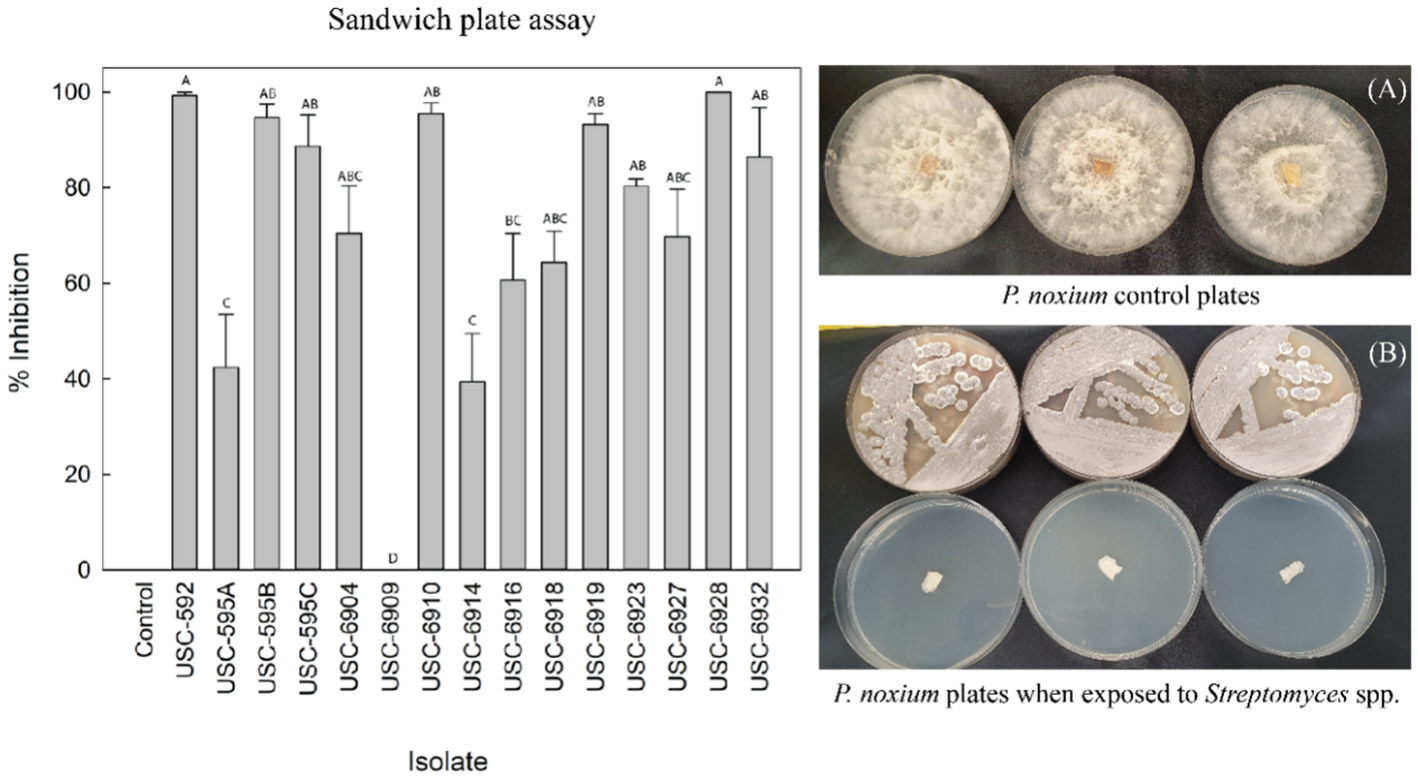
Comparison of root and shoot length and chlorophyll A and B content of the *Arabidopsis thaliana* after 2-week exposure to VOCs produced by the streptomycetes (mean + 1SE). **(A)** Control (only oatmeal agar), **(B)** USC-592, **(C)** USC-595 and **(D)** USC-6928.

Fifteen *Streptomyces* isolates were assessed for antifungal VOC activity against *P. noxium* (F _(14, 30)_ = 13.191, p =<0.001) (**Figure 2**). Fourteen of them displayed antifungal activity from their emitted VOCs, ranging from 39.39%-100% inhibition. Isolate USC-6928 exhibited the strongest activity (100%), followed by USC-592 (99.24%), USC-6910 (95.45%) and USC-595B (94.69%) (**Figure 2**). These four most active strains were selected for further analysis.

### 3.2 Plant promotional activity of the VOCs produced by streptomycetes

**Figure 3:**
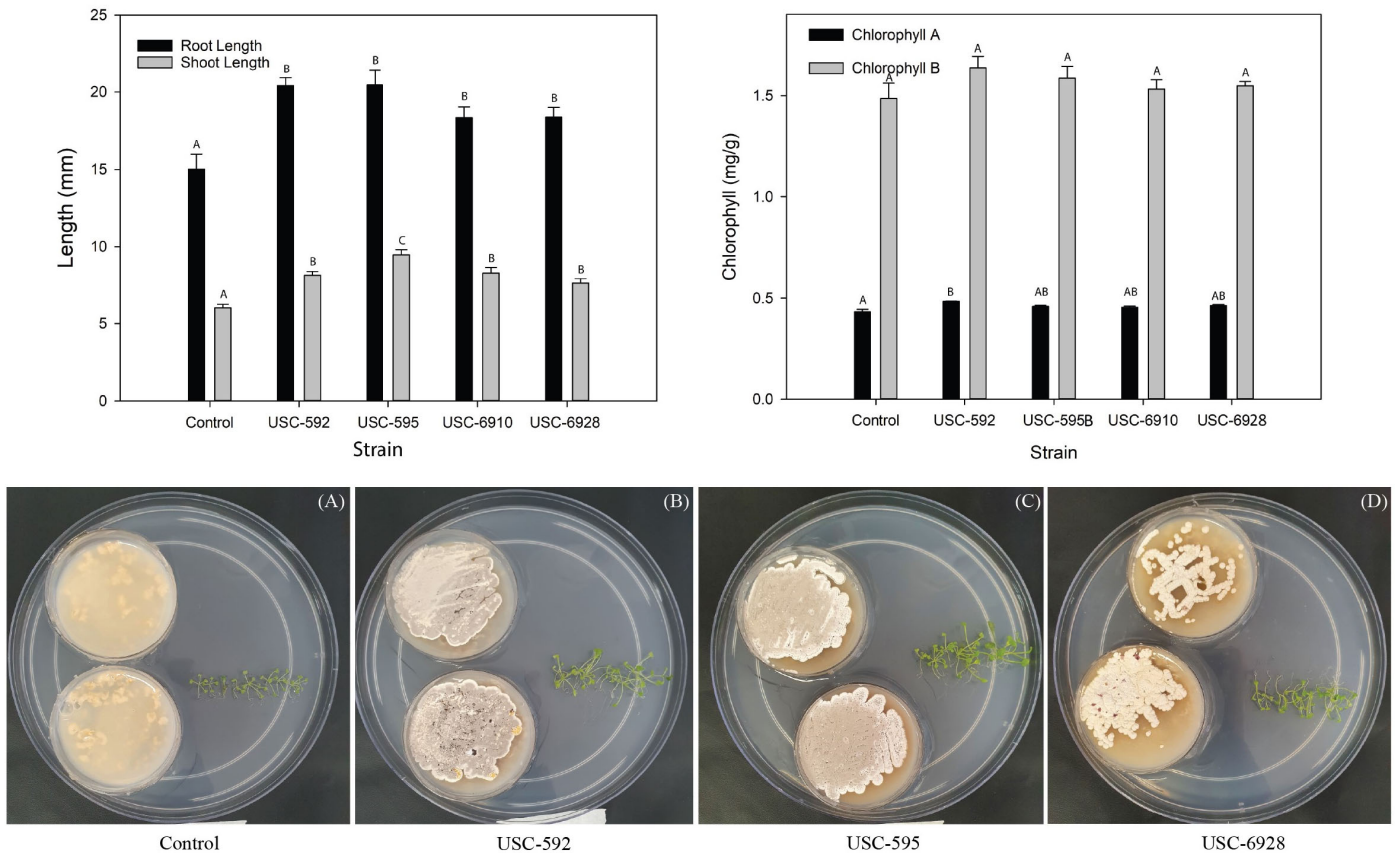
Antifungal VOCs activity detected from the actinomycete isolates against *P. noxium* using the sandwich plate method (mean + 1SE). **(A)** control plates of *P. noxium* without exposure to the VOCs of the actinomycete isolates. **(B)** *P. noxium* plates (bottom plates) when exposed to VOCs produced by USC-6910 (top plates).

The root and shoot length of the *A. thaliana* was measured after 2 weeks of exposure to the VOCs produced by the streptomycete isolates to detect any plant promotional activity (**Figure 3**). A significant difference in growth of the treated samples was found in contrast to the control for the shoot length (F _(4, 20)_ = 5.609, p = 0.003) but not for the root length (F _(4, 20)_ = 1.861, p = 0.157). USC-592 had a 36% increase in root-length and 35% increase in shoot-length. USC-595 had 36% and 57%, USC-6910 had 22% and 37% and USC-6928 had 22% and 26% increase in root and shoot length respectively. No significant differences were found between the treatment samples, except for the isolate USC-595 where the shoot length was found to be 58.3% longer than USC-6928 when effect of the VOCs exposures on the test plant were compared. Chlorophyll A & B were calculated based on the measured wavelengths (**Figure 3**). Significant differences were found between treatment samples and the control for Chlorophyll A (F _(4, 10)_ = 7.157, p = 0.05) but no difference was found for chlorophyll B (F _(4, 10)_ = 2.210, p = 0.141). USC-592 appeared to induce largest increase in the chlorophyll content with an 11.8% increase for chlorophyll A and 10% for chlorophyll B. This was followed by USC-595 with 6.1% and 6.7%, USC-6928 with 7.1% and 4.2%, USC-6910 with 5% and 3.1% increase in chlorophyll content for chlorophyll A and B respectively.

### 3.3 Headspace-solid-phase microextraction-gas chromatography-mass spectrometry analysis of streptomycete VOCs

**Figure 4:**
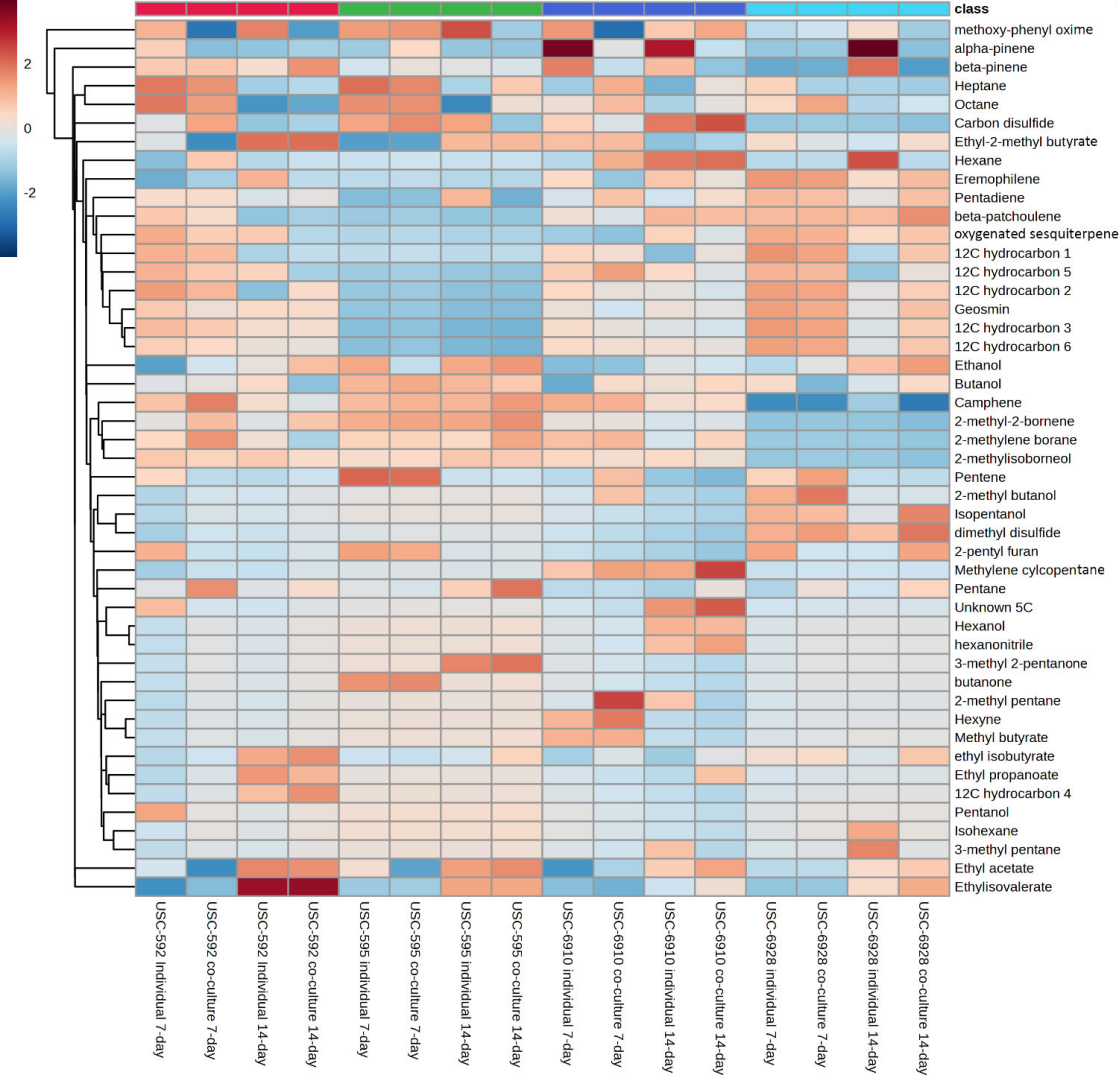
Hierarchical cluster and heat-map analysis of VOC profiles of the streptomycete isolates using Euclidean Distance with arithmetic mean clustering. Columns represent different sample conditions. Rows represent different VOCs (red, high abundance; blue, low abundance).

Identified compounds and their relative peak area percentage were analyzed and a total of 47 VOCs were characterized across a variety of chemical classes. These were: alcohols, terpenoids, esters, ketones and hydrocarbons. Some of the most abundant VOCs were 2-methyl-2-bornene (9.94-41.98%), ethanol (0.82-32.66%), pentene (8.17-31.03%), an unknown 12C hydrocarbon 2 (2.86-26.81%), α-pinene (0.19-20.90), methylene-cyclopentane (2.17-16.75%), methoxyphenol-oxime (0.28-15.60%), pentane (1.15-14.8%) and ethyl isovalerate (0.19-14.65%). Ethanol appears to increase in concentration when the growth period is longer as well as when co-culture conditions were tested. Similarly, methylene cyclopentane increased with time, especially during co-culture conditions used by the isolate USC-6910. α-pinene was also only produced by USC-6910. Pentene appears to only be produced within the 7-day growth period, whereas ethyl-isovalerate seems to be produced only after the 14-day growth period and was especially in a concentrated form by the isolate USC-592. The methoxyphenol-oxime was downregulated under co-culture conditions and pentane was greatly increased under co-culture conditions. Several compounds were observed within the retention times of 11.44-12.01 min that were not able to be unambiguously identified, although the closest matches were all 12C hydrocarbons. Even though unidentified, due to their high abundance, they are worthy of reporting. Isolate USC-595 was the only one found to not produce these unknown compounds. The results from the heat-map (**Figure 4**) show distinct differences in the VOC profiles of the *Streptomyces* spp. under different growth conditions, suggesting that these conditions may impact the production of specific VOCs.

### 3.4 SEM micrographs of *P. noxium* hyphae when exposed to VOCs produced by streptomycete isolate (USC-595B)

**Figure 5:**
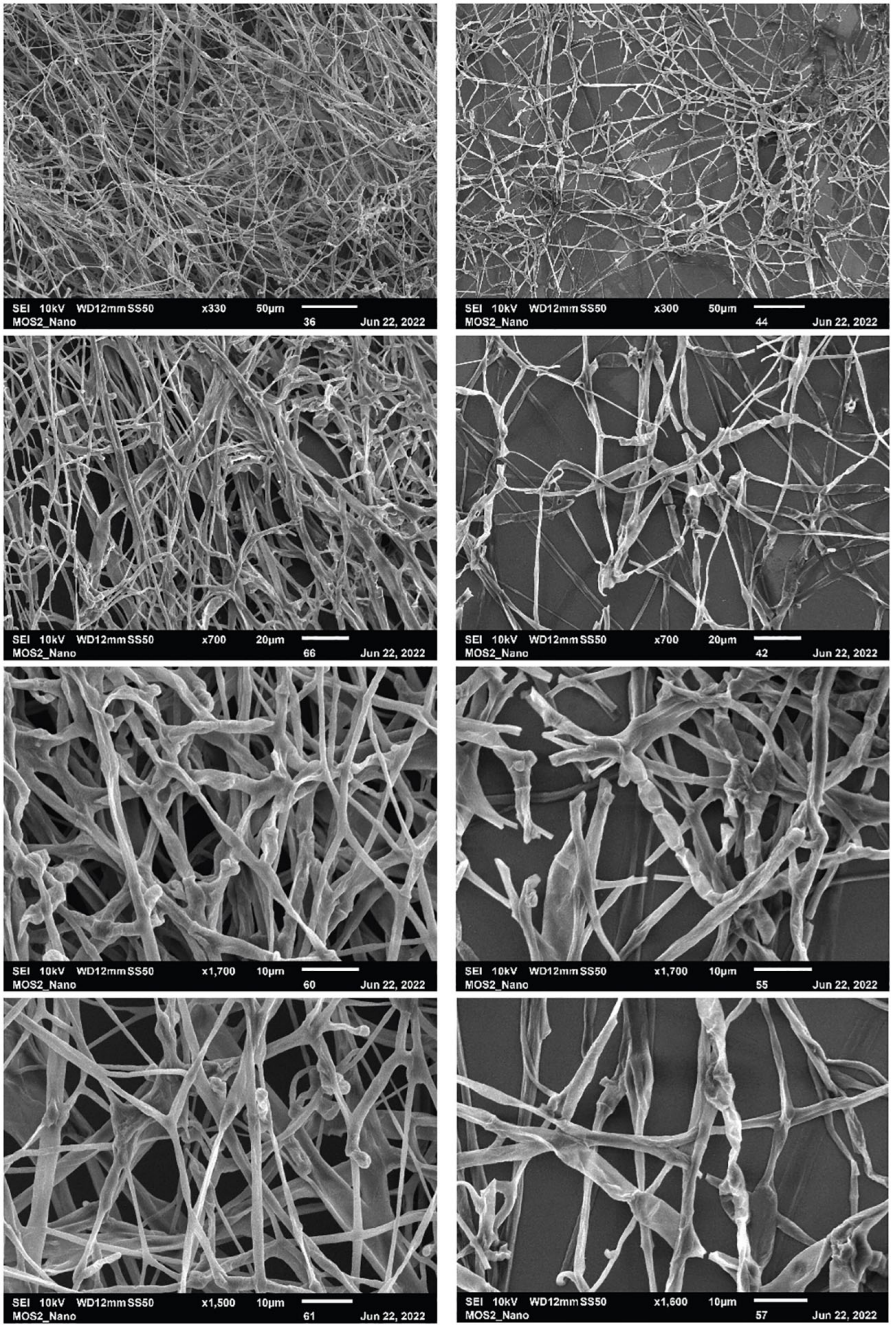
Scanning electron micrographs of *P. noxium* grown in the absence (left-side) and presence (right-side) of the VOCs produced by the streptomycete isolate USC-595B. Scale bar is 50 µm, 20 µm, 10 µm and 10 µm from top to bottom respectively.

The SEM images (**Figure 5**) contrast healthy fungal hyphae (control) with hyphae exposed to VOCs produced by the isolate USC-595. It is apparent from the micrographs that the VOC exposed hyphae have less growth. Within the micrographs, segmentation and sporulation of the hyphae can be observed as well as denaturation and flattening of the hyphal cell wall and many breakages throughout the hyphae of the mycelia.

### 3.5 Indole acetic acid and siderophore production from streptomycetes

Production of indole-3-acetic acid (IAA) was measured for the 15 selected isolates over a period of 7 and 14 days. The streptomycete isolates demonstrated a large amount of IAA production (µg/mL) which varied amongst the isolates and within the different time points (**Table 1**). At day 7, significant differences were observed between the samples and control (F _(14, 30)_ = 19006.197, p =<0.001). USC-595B showed the highest IAA production (43.83 ± 0.94 µg/mL), whilst USC-6910 had the lowest production (5.38 ± 0.05 µg/mL). Day 14 also showed significant differences between the samples and the control (F_(14, 30)_ = 34162.122, p =<0.001). USC-6916 had the highest IAA production (80.64 ± 0.07 µg/mL), while USC-6928 had the lowest production (8.16 ± 0.05 µg/mL). Most isolates increased IAA production with time except for isolates USC-592, USC-595B, USC-595C and USC-6928 that had a decrease in production. USC-6916 had the largest increase in IAA production with a 701% increase from 7-day to 14-day.

**Table 1:** Mean values (± 1SE) values of Indole Acetic Acid (IAA) production from the streptomycete isolates.

Isolate ID	IAA production (µg/mL) at 7 days	IAA production (µg/mL) at 14 days
USC-592	26.66 ± 0.06 ^H^	24.18 ± 0.41 ^F^
USC-595A	8.99 ± 0.05 ^C^	10.53 ± 0.35 ^C^
USC-595B	43.83 ± 0.94 ^L^	30.71 ± 0.57 ^G^
USC-595C	14.17 ± 0.09 ^F^	14.13 ± 0.25 ^D^
USC-6904	10.71 ± 0.14 ^E^	47.63 ± 0.08 ^L^
USC-6909	28.83 ± 0.18 ^I^	32.38 ± 0.39 ^H^
USC-6910	5.38 ± 0.05 ^A^	16.86 ± 0.05 ^E^
USC-6914	9.07 ± 0.12 ^C^	40.2 ± 0.14 ^J^
USC-6916	10.05 ± 0.04 ^D^	80.64 ± 0.07 ^M^
USC-6918	9.27 ± 0.02 ^C^	9.16 ± 0.34 ^B^
USC-6919	8.21 ± 0.51 ^B^	8.34 ± 0.19 ^A^
USC-6923	19.42 ± 0.03 ^G^	45.77 ± 0.07 ^K^
USC-6927	8.20 ± 0.54 ^B^	8.40 ± 0.12 ^A^
USC-6928	30.19 ± 0.32 ^I^	8.16 ± 0.05 ^A^
USC-6932	32.76 ± 0.10 ^J^	36.05 ± 0.13 ^I^

Within a column, values followed by the same letter are not significantly different to each other.

**Figure 6:**
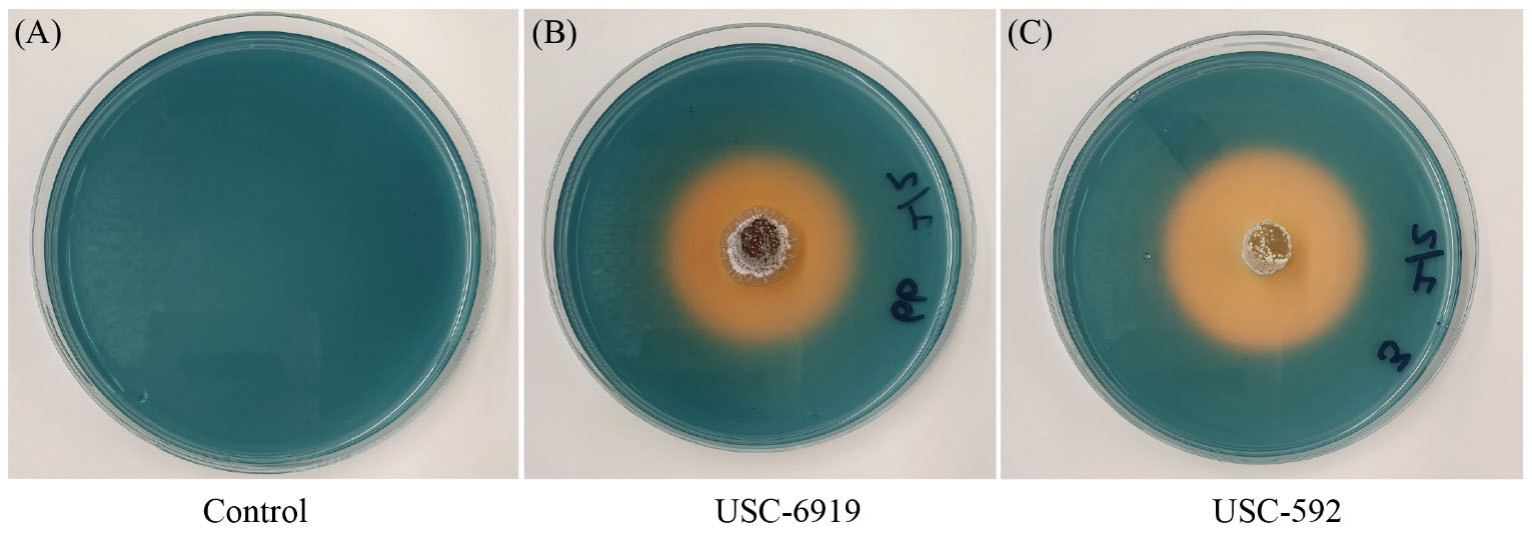
Detection of siderophore production among the streptomycete isolates. Orange clearance zones are indicative of the level of production. **(A)** Control CAS agar plate **(B)** strain USC-592 **(C)** USC-6919.

The siderophore production for the streptomycetes were measured through the appearance of clearance zones around the bacterial colonies (**Figure 6**). The results showed that there were significant differences in siderophore production among the strains tested (F _(14, 30)_ = 87.846, p =<0.001). USC-592 had the largest clearance zone 43 ± 0.82 mm, indicating the highest siderophore production and USC-595C had the smallest clearance zone 20 ± 0.47 mm (**Table 2**).

**Table 2:** Mean (± 1SE) size of clearance zones indicating siderophore production from the streptomycete isolates.

Isolate ID	Clearance zone (mm)*
USC-592	43 ± 0.82 ^G^
USC-595A	24 ± 0.82 ^B^
USC-595B	32 ± 1.70 ^DE^
USC-595C	20 ± 0.47 ^A^
USC-6904	36 ± 1.24 ^F^
USC-6909	29 ± 0.94 ^CD^
USC-6910	34 ± 0.47 ^EF^
USC-6914	36 ± 0.47 ^F^
USC-6916	31 ± 1.24 ^DE^
USC-6918	24 ± 0.47 ^B^
USC-6919	40 ± 0.47 ^G^
USC-6923	24 ± 1.24 ^B^
USC-6927	33 ± 0.47 ^EF^
USC-6928	31 ± 1.24 ^DE^
USC-6932	26 ± 1.25 ^BC^

### 3.6 Chitinase production by streptomycete isolates

**Figure 7:**
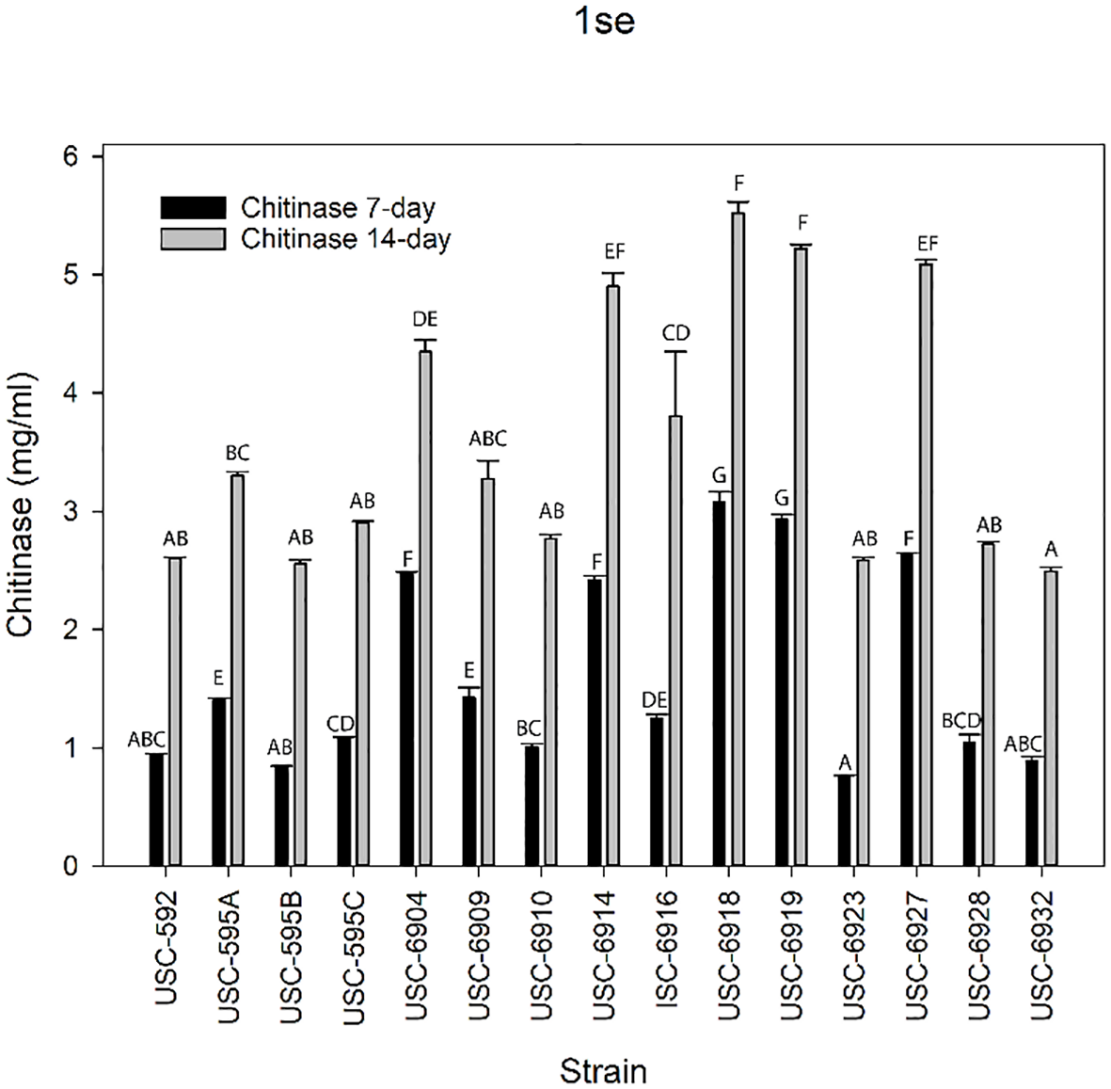
Mean values (+ 1SE) of chitinase production from the termite gut-associated streptomycetes after 7 and 14-days of growth. Letters indicate significant differences according to the Tukey test.

Chitinase production was measured for the streptomycetes using the DNS method to quantify the NAGA production (**Figure 7**). Chitinase activity was observed for all tested isolates at both day 7 (F _(14, 30)_ = 398.366, p =<0.001) and day 14 (F _(14, 30)_ = 51.591, p =<0.001) incubation periods. USC-6918 and USC-6919 displayed the highest activities at day 7 (mean activity 3.08 ± 0.068 mg/mL and 2.93 ± 0.046 mg/mL) and day 14 (5.52 ± 0.094 mg/mL and 5.22 ± 0.035 mg/mL) respectively. The isolates with the lowest chitinase activity at day 14 incubation period were USC-6932 (2.60 ± 0.0319 mg/mL) and USC-6923 (0.89 ± 0.011 mg/mL) for day 7 incubation. Notably, USC-6916 showed the largest increase between incubation periods with a 67% increase in enzyme production.

## 4 Discussion

This study investigated the antifungal activity of the VOCs produced by 15 different streptomycete isolates against *Pyrrhoderma noxium*; a pathogenic fungus that causes significant damage to over 250 plant species. Among the 15 isolates tested, 14 were found to emit bioactive VOCs against *P. noxium* with significant percentages of mycelial growth inhibition. These isolates were previously characterized using 16SrRNA oligonucletotide sequencing (Adra et al., 2023) and the four isolates with the strongest antifungal activity were USC-592 (closest relative of *S. albiaxialis*), USC-595 (closest relative of *S. platensis*), USC-6910 (closest relative of *S. murinus*) and USC-6928 (closest relative of *S. diastaticus*). Other research studies demonstrated that *S. platensis* produced VOCs that effectively reduced disease and pathogens’ mycelial growth in rice, oilseed rape, and strawberries infected with *Sclerotinia sclerotiorum* and *Botrytis cinerea* through consistent fumigation (Liu et al., 2015). However, little research has been conducted on the antifungal VOCs produced by *S. murinus*, *S. albiaxialis*, and *S. diastaticus* although, other studies have shown non-volatile antifungal activity of *S. murinus* against *Plectosporium tabacinum* (Wan et al., 2008). *S. diastaticus* is also known to produce butyrolactol A, an antifungal polyketide (Youssef et al., 2001; Wan et al., 2008). Some antifungal activity against *Saccharomyces cerevisiae* has also been reported from *S. albiaxalis* (Sarmiento-Vizcaíno et al., 2022). Overall, USC-595 which was identified as a closest relative of *S. platensis* (Adra et al., 2023b) produced the strongest evidence supporting its antifungal potency of VOCs, however, no known report was found for the use of streptomycetes’ VOCs against *P. noxium.* Therefore, these four selected isolates might be ideal candidates and provide a groundwork for the first-time use of streptomycetes against the fungal pathogen *P. noxium*.

Furthermore, the capacities of the *Streptomyces* spp. to produce VOCs that are associated with the promotion of plant growth was also observed. *Streptomyces* isolates significantly promoted the *in vitro* growth of *A. thaliana* seedlings, especially the growth in the roots (22-36%) and shoots (26-57%). Whilst chlorophyll content did not appear to have much difference between the control and treated samples, USC-592 demonstrated the highest variation in chlorophyll content (10-11.8%). These findings are not unexpected as prior studies have also reported minimal differences in chlorophyll content of model plants in the presence of streptomycetes. For instance, in bean seedlings, differences up to 10% were reported in chlorophyll content (Pérez-Corral et al., 2022). In consideration of the observed antifungal and plant promotional activity, these VOCs serve as potential leads in the development of biocontrol agents that could be utilized as bio-stimulants or biofumigants in agriculture.

From the HS-SPME-GC-MS analysis, 47 major VOCs produced by the *Streptomyces* were detected and identified. Among them, geosmin and 2-methyl-2-bornene (a precursor for 2-methylisoborneol) were found at high concentrations. No published data was found for 2-methyl-2-bornene’s antifungal activity, however, geosmin has been found to inhibit the growth of *Aspergillus parasiticus* and *Aspergillus flavus* (Boukaew and Prasertsan, 2020). 2-methylisoborneol was present in all of the streptomycete isolates except the USC-6928. This compound has previously demonstrated antifungal activity against *A. flavus*, *Aspergillus orchraceus*, *Aspergillus niger* and *Penicillium citrinum* (Wang et al., 2013). Similarly, 2-methylbutyrate inhibits conidial germination and mycelial growth of *A. flavus* and *A. parasiticus* (Lyu et al., 2020) and was present in all tested isolates and produced most by the isolate USC-592. Ethyl isovalerate was found in all isolates but only after the 14-day growth period. This compound was reported to influence primary root architecture and the expression of auxin and jasmonic acid-response genes, although these were from rhizobacteria, mainly *Bacillus* and *Brevibacillus* spp (Gamboa-Becerra et al., 2021; Rani et al., 2023). Methoxyphenyl-oxime found in this study has also been related to plant promotion activity. It was produced in most concentrated amount by the isolate USC-592, although only when in monoculture, not paired by the pathogen. The isolate USC-595 whereas produced it in both mono and co-culture conditions but compound concentration decreased dramatically in co-culture. Many important auxins and bioactive metabolites including volatiles, are produced from oximes (Sørensen et al., 2018) and play roles in growth regulation, plant defense and communication. Methoxyphenyl-oxime has been isolated from *S. pratensis* and had selective antibacterial activity but no effect on *S. cerevisiae* (Barghouthi et al., 2017). Thus, it is likely to be more beneficial for plant promotion than plant defense against *P. noxium*. The monoterpenes α- and β-pinene which were found in all isolates, are known plant growth stimulants and β-pinene has antifungal activity against *Aspergillus ochraceus* (Yang et al., 2018) and *A. flavus* (Yang et al., 2019). Methylene cyclopentane was found to be produced only by the isolate USC-6910 and increased in concentration dramatically in co-culture condition; suggesting it likely plays a role in fungal antagonism and may be worth investigating further as a potential biocontrol product. It is an important building block for complex structures, for example, 2-amino-4-methylene-cyclopentane-1-carboxylic acid, also known as icofungipen, has shown *in vitro* activity against several *Candida* spp (Ziegelbauer et al., 1998). Other VOCs that may not be considered major compounds due to their relatively low abundance but are worth noting due to their activity include dimethyl disulfide which was only found to be produced by the USC-6928 and increased in concentration under co-culture conditions. It has also been reported to display antifungal activity against *Fusarium moniliforme* (Wang et al., 2013), *Caralluma fimbriata* (Gong et al., 2022) and *A. ochraceus* (Yang et al., 2018). Key VOCs identified in this study might therefore be responsible for the observed antifungal activity against the *P. noxium*, as well as the plant promotional activity towards *A. thaliana*. These results support the capability of termite-gut associated streptomycetes as potential biological control agents by demonstrating the antifungal activities from the VOC they produce.

In addition, it is also evident that the observed IAA and siderophore productions also contribute to these isolates’ ability to promote plant growth. These compounds play an important role within the auxin family of plant hormones (Myo et al., 2019) by assisting plant development and physiological processes (Kaur and Manhas, 2022) and have widely been reported in the genus *Streptomyces* (Pérez-Corral et al., 2022). Overall, isolates USC-6916 and USC-6928 demonstrated the highest quantities of IAA at 79.57 ± 0.07 µg/mL and 74.80 ± 0.05 µg/mL respectively. Generally, reports from other studies suggest a range between 20-136 µg/L and some strains produce very low amounts of IAA (around 6-7 µ/L) (Pérez-Corral et al., 2022). A study conducted by Kaur and Manhas (Kaur and Manhas, 2022) assessed 60 *Streptomyces* isolates for IAA production and observed a range between 2.33-121.92 µg/mL. IAA-producing microorganisms have been proposed as bio-fertilisers for the large-scale exploitation of crops and results from this study indicate that termite gut-associated streptomycetes may be suitable candidates. Additionally, iron is an important essential element for photosynthesis, respiration, and DNA replication in plants. However, iron’s poor solubility makes it not readily bioavailable for organisms. Therefore, one approach for the acquisition of ferric iron is the biosynthesis of siderophores (Sharma et al., 2020). All *Streptomyces* isolates (n=15) in this study displayed the ability to produce siderophores with USC-592 having the largest clearance zone at 43 ± 0.82 mm. One study tested a known plant-beneficial strain, *S. alfalfae*, for siderophore production using the same CAS medium assay and observed a clearance zone of 15 ± 1.02 mm after 10 days (Niu et al., 2022). Another study assessed plant growth-promoting rhizobacteria (PGPR) for siderophore production and found clearance zones ranging from 26-75 mm (Horstmann et al., 2020). Most of the strains used within this study also fell within this range, making them comparable to these PGPR. The *Streptomyces* isolates in this study also demonstrated the ability to produce chitinase. Chitinase is an important hydrolytic enzyme that aids in the lysis of chitin. It supports their ability to obtain nutrients through degradation of environmental chitin which also includes the fungal cell wall (Abo-Zaid et al., 2021; Meena et al., 2022). A study investigated the chitinase production from a streptomycete isolated from agricultural farmland over time and found it peaked at 72 hours with 8 mg/mL before it started declining (Sani et al., 2021). The enzyme produced by the *Streptomyces* isolates used in this study increased in production from the 7-day incubation period to the 14-day incubation with USC-6918 and USC-6919 reaching 5.52 ± 0.094 mg/mL and 5.22 ± 0.046 mg/mL respectively. These results demonstrate additional mechanisms of biological control and further support the premise that termite gut-associated streptomycetes have potential in promoting plant growth as well as inhibiting fungal pathogens. Thus, making them ideal candidates as biological control agents and are worth investigating further to control *P. noxium*. The importance of exploring rare and previously unknown actinomycete taxa has been communicated by many researchers including the termite-gut associated ones in terms of discovery of novel bioactive compounds (Kurtböke et al., 2014; Kurtböke, 2024; Romero et al., 2024). During the adaptation of such extreme environments, microorganisms can produce additional or different compounds which might be required to adapt the new environment thus providing different chemical structured compounds. Findings in this study also confirms that such actinomycetes can offer more for biotechnological and biocontrol applications.

## 5 Conclusions

*Streptomyces* isolates USC-592, USC-595, USC-6910 and USC-6928 had the strongest antifungal VOC production. The isolates also demonstrated effective plant promotional activity through increase of root and shoot length of *A. thaliana*. Of the forty-seven identified VOCs, four key VOCs are known to greatly contribute to plant promotion and six other compounds are known to have antifungal activity against various other fungal phytopathogens. Further research could focus on the application of VOCs of these streptomycetes in crop protection and plant growth promotion in agricultural systems. It could also include understanding of the mechanisms behind VOC production by streptomycetes and the influence environmental factors may have on their specific VOCs profiles.

## Data availability statement

The raw data supporting the conclusions of this article will be made available by the authors, without undue reservation.

## Author contributions

CA: Conceptualization, Writing – review & editing, Formal Analysis, Investigation, Methodology, Writing – original draft. HP: Conceptualization, Writing – review & editing, Formal Analysis, Investigation, Methodology. KF: Resources, Writing – review & editing. RT: Resources, Writing – review & editing. RH: Resources, Writing – review & editing, Investigation, Methodology. DK: Resources, Writing – review & editing, Conceptualization, Funding acquisition, Project administration, Supervision.
